# WECHAT SOCIAL MEDIA USE AND DEPRESSION AMONG CHINESE OLDER ADULTS

**DOI:** 10.1093/geroni/igad104.2156

**Published:** 2023-12-21

**Authors:** Wan Wen, Shannon Mejia

**Affiliations:** University of Illinois Urbana-Champaign, Urbana, Illinois, United States; University of Illinois Urbana-Champaign, Chanpaign, Illinois, United States

## Abstract

Impairments in health and mobility, the loss of a partner, and geographic relocation increase the risk of social isolation and depression in older adulthood. As the world’s most populous country, China faces a rapidly aging population, and Chinese society recognizes the potential of the Internet as a measure to reduce depression and promote well-being among older adults. WeChat is the most used social media among Chinese older adults, who use it to connect with social partners and exchange information by reading and posting messages. However, previous research only identified a correlation between WeChat use and lower depressive symptoms but failed to uncover the causality between them. This paper used data from the 2018 China Health and Retirement Longitudinal Study and adopted an instrumental variable (IV) and the two-stage least squares model to strengthen causal inference. The principle is using an IV, which is correlated with WeChat use but not influenced by depressive symptoms, to replace WeChat use in regression. There were 1,680 participants (mean age 73.03, 55% male), 3% of whom reported use of WeChat in the past two years. Regression results suggested that WeChat use did not lead to lower levels of depression among older adults; instead, only non-depressed older adults would use WeChat. Further analysis showed WeChat use could promote older adults’ communication with family members and social activity engagement. Although there is no solid evidence of WeChat’s effect on depression, it does provide some benefits and still holds the potential to promote the well-being of older adults.

